# A Sexual Ornament in Chickens Is Affected by Pleiotropic Alleles at *HAO1* and *BMP2*, Selected during Domestication

**DOI:** 10.1371/journal.pgen.1002914

**Published:** 2012-08-30

**Authors:** Martin Johnsson, Ida Gustafson, Carl-Johan Rubin, Anna-Stina Sahlqvist, Kenneth B. Jonsson, Susanne Kerje, Olov Ekwall, Olle Kämpe, Leif Andersson, Per Jensen, Dominic Wright

**Affiliations:** 1 IFM Biology, AVIAN Behavioural Genomics and Physiology Group, Linköping University, Linköping, Sweden; 2 Department of Medical Biochemistry and Microbiology, BMC, Uppsala University, Uppsala, Sweden; 3 Department of Medical Sciences, The Research Group of Autoimmunity, Akademiska Sjukhuset, Uppsala University, Uppsala, Sweden; 4 Department of Surgical Sciences, Orthopaedics, Akademiska Sjukhuset, Uppsala University, Uppsala, Sweden; 5 Department of Rheumatology and Inflammation Research, Institute of Medicine, The Sahlgrenska Academy, Gothenburg, Sweden; Stanford University School of Medicine, United States of America

## Abstract

Domestication is one of the strongest forms of short-term, directional selection. Although selection is typically only exerted on one or a few target traits, domestication can lead to numerous changes in many seemingly unrelated phenotypes. It is unknown whether such correlated responses are due to pleiotropy or linkage between separate genetic architectures. Using three separate intercrosses between wild and domestic chickens, a locus affecting comb mass (a sexual ornament in the chicken) and several fitness traits (primarily medullary bone allocation and fecundity) was identified. This locus contains two tightly-linked genes, *BMP2* and *HAO1*, which together produce the range of pleiotropic effects seen. This study demonstrates the importance of pleiotropy (or extremely close linkage) in domestication. The nature of this pleiotropy also provides insights into how this sexual ornament could be maintained in wild populations.

## Author Summary

The genetic analysis of phenotypes and the identification of the causative underlying genes remain central to molecular and evolutionary biology. By utilizing the domestication process, it is possible to exploit the large differences between domesticated animals and their wild counterparts to study both this and the mechanism of domestication itself. Domestication has been central to the advent of modern civilization; and yet, despite domesticated animals displaying similar adaptations in morphology, physiology, and behaviour, the genetic basis of these changes are unknown. In addition, though sexual selection theory has been the subject of a vast amount of study, very little is known about which genes are underpinning such traits. We have generated multiple intercrosses and advanced intercrosses based on wild-derived and domestic chickens to fine-map genomic regions affecting a sexual ornament. These regions have been over-laid with putative selective sweeps identified in domestic chickens and found to be significantly associated with them. By using expression QTL analysis, we show that two genes in one region, *HAO1* and *BMP2*, are controlling multiple aspects of the domestication phenotype, from a sexual ornament to multiple life history traits. This demonstrates the importance of pleiotropy (or extremely close linkage) in controlling these genetic changes.

## Introduction

Domestication is the strongest form of short term, highly directional selection known to man. Darwin himself cited domestication as a model for evolution [1]. The domestication process itself is associated with a whole raft of changes in phenotype, despite intentional selection on only a few traits. For example, simultaneous changes in colour, skull shape, and behaviour [2]–[4] are often observed in domestic populations, and can emerge even when selection is limited to tameness [5], [6]. The genetic mechanisms affecting such correlated changes are largely unknown. Pleiotropy, where one allele affects multiple traits, would be one potential mechanism [5], [6]. Alternatively, traits may be linked at a genetic level, but with separate genetic architectures [7], [8].

Of the relatively few domestic animals, the chicken is one of the most viable as a research animal. A combination of small size, rapid generation times, small genome size (∼1.09 Gb) [9] and high recombination (350 kb/cM on average) [10], along with extreme changes in phenotype (focused on production traits, namely egg production and increased growth and overall size) due to domestication make it particularly amenable as a model organism. As well as an increase in production traits, modern domestic chickens also show a large increase in relative comb size, a sexually selected ornament, and bone production [8].

**Figure 1:**
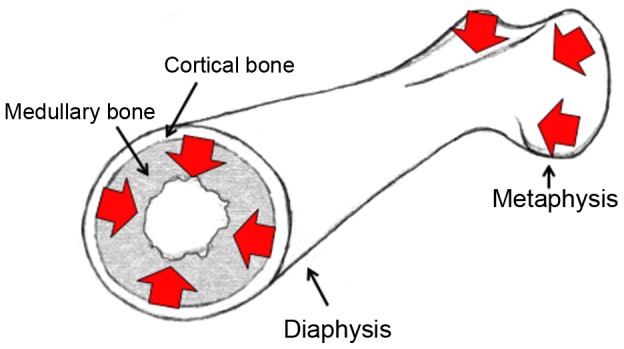
Cartoon of a female femoral bone. The location of medullary and cortical bone is highlighted, as well as the metaphysis and diaphysis. The flow of calcium in egg-laying females is indicated with red arrows, illustrating the transfer of calcium from the metaphyses to the diaphysis, and from the hard cortical bone to the soft medullary bone.

The comb of the chicken is used to base mating decisions on by both males and females in wild-derived populations [11], [12]. In males it is an indicator of social rank, with females actively soliciting matings from males with larger combs [13], [14], as well as also correlating with bone mass [8]. In females it is indicative of greater reproductive potential, through an increase in egg production [8], [15]. In turn, egg production is highly dependent on bone morphology in the chicken, with one of the principal limitations to egg production being calcium deposition. Calcium is stripped from the hard outer cortical bone and transferred into the soft, inner medullary and trabecular bone and from there mobilised to the egg shell [16]. Similarly, calcium is also mobilised away from the ends of the bones (the metaphyses) and into the central part (the diaphysis) during egg-laying periods, where it is then more easily mobilised into medullary bone (see Figure 1). The genetic architectures for comb mass, egg production and bone allocation (important for egg production) have all been shown to overlap in the chicken [8]. Therefore fine-scale quantitative trait loci (QTL) mapping and expression quantitative trait locus (eQTL) mapping of these separate traits (comb mass, bone allocation and egg production) should allow the assessment of the importance of pleiotropy in both domestication and a sexual ornament. The bi-sexual expression of the comb in both males and females makes the comb somewhat unusual as a sexual ornament, with male-only effects of ornaments more often considered [17], and also makes the genetic analysis of this ornament in particular of greater relevance.

Here we present the identification of a two-gene block controlling multiple phenotypic and fitness traits. This block was identified due to its extreme effect on comb mass, with its effects on bone allocation and fecundity then ascertained. A total of three different intercrosses, based on two different Layer populations and two different Red Junglefowl populations, were used to QTL map these traits. The two Layer populations consisted of two White Leghorn (WL) layer breeds (termed Obese strain (OS) and Line 13 (L13) hereafter), whilst these were crossed with two different populations of Red Junglefowl (RJF) chickens (the wild ancestor to the domestic chicken). Both Layer populations show a marked increase in comb mass and bone allocation, as compared to the RJF populations. The L13 cross was in addition expanded from an F_2_ population to an F_8_ advanced intercross. Therefore a F_2_L13, a F_8_L13 and a F_2_OS cross were all utilised. Fine-scale resolution and replication was achieved by over-laying the multiple QTL signals from the different crosses in combination with the small (40 kb) signatures of selected loci that have been previously identified in the domestic chicken using extensive resequencing [18]. Finally, eQTL analysis was performed using specific tissue types from the F_8_L13 cross to further test the causal association of genes affecting the sexual ornament of comb mass and the fecundity and fitness trait of medullary bone allocation.

## Results

### Comb Mass Architecture

**Figure 2:**
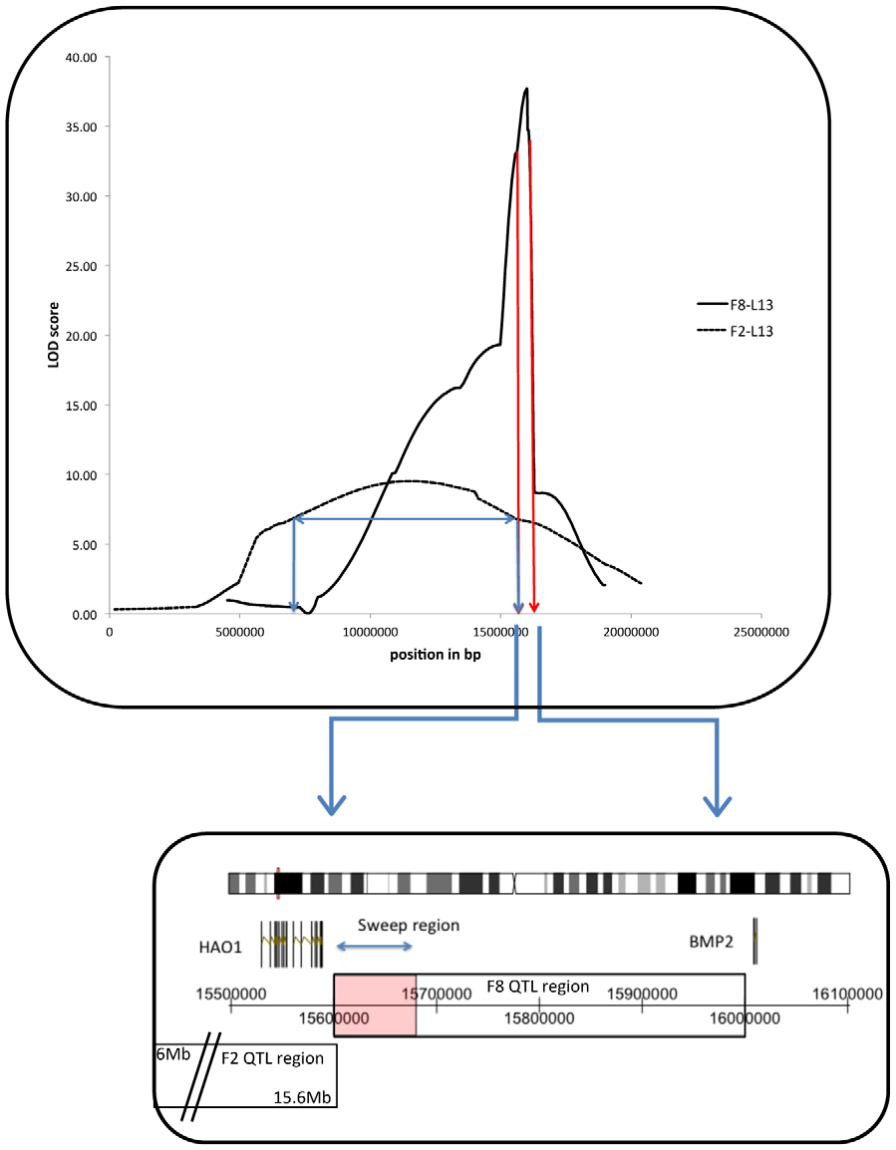
Chromosome 3 QTL peaks and confidence intervals (C.I.) from the F_2_-L13 (blue line) and F_8_-L13 (dark red line) crosses. In the case of the F_2_-L13, the horizontal arrow indicates the 1.8 LOD drop C.I. of the QTL position. The location of the F_8_-L13 C.I. has been expanded below the graph to show the location of the two candidate genes and the selective sweep, and the overlap with the F_2_-L13 C.I.

The genetic architecture for comb mass from all three cross populations was shown to be principally restricted to six distinct regions in the genome (see Table S1), with a strongly significant degree of overlap between the different crosses (clustering test with 10000 permutations, P = 0.0016, see Tables S1 and S2 and Figure S1). In five of the six clusters, all QTL in the cluster had the same direction of effect (i.e. the allele conferring the larger effect was always based in the same population), adding strength that these were independent replicates of the same QTL. All of these alleles that were associated with larger combs derived from the WL strain, with the exception of a locus on chromosome one that showed transgressive segregation. Even here, the same pattern of transgressive segregation was mirrored in all crosses, once again reinforcing the idea of independent replication. The largest effect QTL in the F_2_L13 and F_8_L13 crosses was on chromosome three, with an additive effect of 4.3 grams and 10.9 grams, respectively, and LOD scores of 10 and 38 (R^2^ = 11% in F_2_L13 males, 30% in F_8_L13 males and 10% in F_8_L13 females). The confidence interval (C.I., calculated with a 1.8 LOD drop) for the F_2_L13 cross was from 6.6 Mb to 15.6 Mb, whereas the C.I. for the F_8_L13 was from 15.6 Mb to 16.0 Mb. The two intervals therefore only overlap at 15.6 Mb (see Figure 2), with the two markers defining these regions in the F_2_L13 and F_8_L13 crosses within 100 bp of one another. Of the other significant QTL identified in the F_8_L13, the one with the second smallest C.I. was located on chromosome 8, from 19.6–21.6 Mb with a LOD score of 10. In both the case of the chromosome three and eight QTL regions, although the loci were found in the F_2_OS cross, the size of the overall C.I. was not reduced.

### Comb QTL and Selective Sweep Overlap

Strong selection has been hypothesised to leave signatures of selection (selective sweeps) identifiable through linkage disequilibrium (LD) blocks present in the genome. The identification of such sweeps is becoming more common, though as yet all the successes of gene identification that have occurred through this approach have been ones of major effect, more akin to a straight Mendelian than a quantitative trait (but see [19]). Indeed, there is a great deal of debate as to whether such sweeps are even relevant for quantitative traits, or whether polygenic adaptation (small changes in gene frequency) is a more viable mechanism [20]. Despite this, resequencing of domestic and wild strains has yielded a number of putative selective sweeps in the chicken [18], each ∼40 Kb in size. These regions were overlaid with the six overlapping C.I. from the comb QTL studies, with five of the six overlap regions containing one or more sweeps, a significant enrichment (clustering test, P = 2×10^−4^). All of these were either layer-specific (i.e. regions fixed in all layers) or all-domestic (i.e. regions fixed in both layers and broilers), see Table S2. The largest QTL on chromosome 3 contained a selective sweep, located at 15.6 Mb, at precisely the region where the overlap between the F_2_L13 and F_8_L13 crosses occurred. The chromosome 8 QTL also contained a selective sweep present at the QTL peak, at 19.7 Mb.

### Expression of Candidate Genes in Comb Mass

The chromosome 3 QTL C.I. contains 2 reference genes and a single selective sweep. This sweep contains the gene *hydroxyacid oxidase (HAO1*), whilst the gene *bone morphogenetic protein 2 (BMP2*) is adjacent to it (see Figure 2). Gene expression analysis was performed for the two putative causative genes on chromosome 3 using material from the comb base of 41 male F_8_L13 birds and from the diaphyseal medullary bone of 20 F_8_L13 female birds. Medullary bone tissue was used as analysis of medullary area showed a suggestive QTL co-localising at the identical location as to the comb mass QTL, with its peak also at 15.6 Mb. Both genes were found to be differentially expressed between RJF and WL alleles in comb base tissue (*HAO1* P = 2×10^−6^, *BMP2* P = 0.02), and the expression levels were strongly positively correlated with comb mass in both cases (*HAO1* P = 0.0001, *BMP2* P = 0.002, see Table 1.

**Table 1:** General Linear Model results for gene expression and QTL effect on comb mass.

Model - comb base	Batch	Bodyweight	QTL genotype	HAO1 expression	BMP2 expression	Adj R^2^	AIC
QTL only	4.3/0.0001	3.5/0.001	5.7/1.8×10-6	-	-	0.56	289.0
HAO1	0.05/0.96	1.0/0.35	-	4.4/0.0001	-	0.4	300.6
QTL+HAO1	2.0/0.06	3.0/0.004	3.1/0.003	1.6/0.12	-	0.58	288.2
BMP2	2.5/0.02	2.0/0.05	-	-	3.4/0.002	0.31	306.4
QTL+BMP2	4/0.0003	4.2/0.0002	4.8/3.4×10-5	-	2.2/0.04	0.6	285.9
HAO1+BMP2	0.05/0.96	2.4/0.02	-	4/0.0003	3/0.005	0.51	293.2
QTL+HAO1+BMP2	1.7/0.11	3.8/0.0006	2.5/0.02	1.8/0.09	2.3/0.03	0.62	284.2

All variables were modelled on comb mass, with the t-statistic and p-value shown for each variable included in each of the models (shown as t-statistic/p-value). ‘-’ is used to indicate where a variable is not included in that particular model. AIC stands for the Akaike Information Criterion of each model. A full description of each model is given in the methods section.

A test to distinguish linkage disequilibrium (LD) from true causality was conducted using expression as a covariate in the genotype/phenotype model [21]. Theoretically, if the QTL effect on the phenotype drops in significance when the gene expression covariate is included this should indicate the gene in question is causal to the QTL [21], [22], as the quantitative trait transcript (QTT) will explain more of the variance previously accounted by the genotype factor. In the case of *HAO1* this occurs, with the variance explained by genotype (R^2^) falling from 44% to ∼18% (P = 2×10^−6^ to P = 0.003) with this covariate inclusion, with the AIC for the model falling from 289 to 288.2, see Table 1. *BMP2* is significantly correlated with comb mass when placed as a covariate in a model already containing genotype (P = 0.04), and genotype significance also decreases with this covariate inclusion (P = 1.8×10^−6^ to P = 3.4×10^−5^, see Table 1, with the R^2^ falling from 44% to 29%), whilst the AIC for the model decreases from 289 to 285.9. Although the principal effect on comb mass therefore appears to be coming from *HAO1* expression, *BMP2* also appears to have an effect. A model including both *HAO1* and *BMP2* gene expression levels explains more of the variation present in comb mass, the R^2^ rising from 40% to 51% and the AIC decreasing from 300.6 to 293.2, compared to the model with only *HAO1* present, with both *HAO1* and *BMP2* having significant effects on comb mass in the model (*HAO1* P = 0.0003, *BMP2* P = 0.005). The model with *HAO1, BMP2* and the QTL effect had the lowest AIC, 284.2, and the highest R^2^, 62%, see Table 1. This demonstrates that both genes are important for determining comb mass, between them explaining the majority of the variation in comb size in the sample used for comb base gene expression.

Associations between a QTL locus, the expression of a gene and a complex trait can be used to try model the path of the QTL effect [23]. Using this idea, both *HAO1* and *BMP2* expression levels can be modelled on all the QTL for comb mass detected in the F_8_L13 cross. This analysis can indicate if the other detected QTL are acting through the same *HAO1*/*BMP2* pathway as the chromosome 3 QTL, though with the caveat that such tests are still in essence correlative and not a substitute for functional assays. Three QTL in a combined model explain more of *HAO1* expression than the chromosome 3 locus in isolation (chr1@35.5 Mb P = 0.03, chr1@172 Mb P = 0.02, chr3@16 Mb P = 2×10^−6^, R^2^ rose from 63% to 71% with all three QTL in the model with the AIC decreasing from −401.1 to −408.8). Similarly, *BMP2* expression is correlated with a total of three QTL in a combined model, two of which are interacting (chr1@35.5 Mb P = 0.02, chr1@35.5 Mb×chr5@32.7 Mb P = 0.03, chr3@16 Mb P = 0.03, R^2^ rose from 32% to 50% with all three QTL in the model, with the AIC decreasing from −126.8 to −134). It therefore appears that both *HAO1* and *BMP2* are determining comb mass, functioning on two separate pathways. The first one involves *HAO1* (and therefore the chr3 QTL), with the QTL on chromosome 1 at 35.5 Mb and 172 Mb are also part of this pathway. The second, involving *BMP2*, involves the chromosome 3 QTL again, with the QTL on chromosome 1@35.5 Mb and chromosome 5@32.7 Mb also involved in this pathway.

### Comb Mass and Bone Allocation

**Figure 3:**
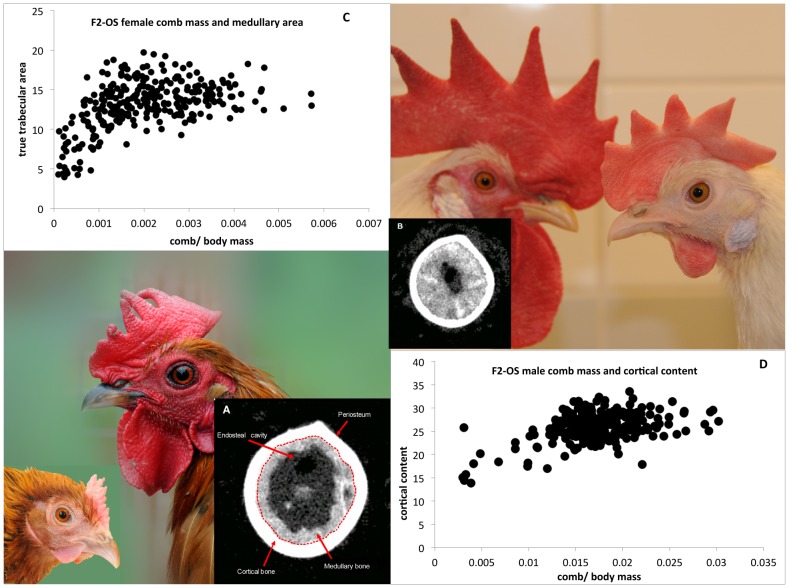
Illustration of male and female RJF (lower left) and WL (upper right) comb size. CT pictures of two diaphyseal bone sections are shown in (A) and (B), with the cortical and medullary bone marked. The correlation between female comb mass relative to body mass and diaphyseal trabecular area shown in (C), and the correlation between relative male comb mass and cortical content is shown in (D).

While comb mass is known to be an indicator of reproductive capacity [8], [15], the links between bone allocation and comb mass are less known [8], though given the close relationship between egg production and bone allocation, some correlation may be expected. Therefore we measured metaphyseal and diaphyseal (see Figure 1, Figure 3a and 3b) bone morphometrics in both the F_2_OS and F_8_L13 mapping populations, using a Computerised Tomography (pQCT) machine (see Materials and methods), and then modelled these bone characteristics on comb mass. Results of the combined General Linear Models are shown in Table S3 and Figure 3. Females with larger combs deposit more calcium in the diaphysis where it is more easily mobilised into the eggshell. This is shown by diaphyseal cortical bone measures being positively correlated with comb mass in both the F_2_OS and F_8_L13, whereas metaphyseal total bone density is negatively correlated in the F_2_OS, and metaphyseal medullary density is negatively correlated in the F_8_L13. Females with larger combs are also producing more of the diaphyseal medullary bone required for egg production. This is indicated by diaphyseal medullary area being positively correlated with comb mass in the F_2_OS (see Figure 3) and diaphyseal medullary density being positively correlated and metaphyseal medullary density negatively correlated with comb mass in the F_8_L13. In males, metaphyseal medullary area and density and diaphyseal cortical content are also positive predictors of comb size, once again showing that males with larger combs are more likely to have greater overall bone density and strength (see Figure 3).

A QTL for medullary bone was also identified at the same locus that effected comb mass on chromosome 3. *HAO1* and *BMP2* were therefore also tested as potentially causative loci for effecting medullary bone. The results for medullary bone similarly indicate a greater effect of *HAO1* rather than *BMP2* on medullary bone area, though the causal tests themselves are less conclusive. When *HAO1* is included in the QTL model as a covariate, the variance explained by QTL genotype increases from R^2^ = 14% to 27% (P = 0.02 to 0.002), with *HAO1* significant in this model (P = 0.03, R^2^ = 10%), whilst the AIC decreases from 135.5 to 131.4, see Table 2. Comb mass was negatively correlated with medullary area (this mirrors the correlation seen between comb and medullary bone in the F_8_L13 cross, where medullary density was more strongly and positively correlated with comb mass). Once again, *BMP2* expression has less effect on QTL genotype, with the variance explained by genotype unchanging (R^2^ = 14% to 17%), and the AIC increasing from 135.5 to 136.3, and *BMP2* expression itself is also not significant (P = 0.35). Expression levels of *BMP2* and *HAO1* in bone tissue were found to be correlated with several related relevant bone strength and fecundity phenotypes, see Table 3. *HAO1* was positively correlated with an increase in egg production (t = 2.8, P = 0.02), a lack of broodiness (egg incubation behaviour, see Materials and methods, t = −2.4, P = 0.03), and an increase in the number of eggs produced (t = 2.6, P = 0.02). *BMP2* expression was correlated with an increase in total density of the diaphyseal endosteal cavity (t = 2.4, P = 0.03) and a decrease in diaphyseal endosteal cavity area (t = −2.2, P = 0.05). It therefore appears that the two linked genes have a pleiotropic effect between them, affecting comb mass, egg production, medullary bone area, and total area and density of the endosteal cavity.

**Table 2:** General Linear Model results for gene expression and QTL effect on bone mass.

Model - bone mass	Bodyweight	QTL genotype	HAO1 expression	BMP2 expression	Adjusted R^2^	AIC
QTL only	5.4/7.8×10-5	2.6/0.02	-	-	0.61	135.5
HAO1	3.9/0.001	-	0.2/0.83	-	0.44	142.2
QTL+HAO1	6.6/1.2×10-5	3.8/0.002	-2.4/0.03	-	0.71	131.4
BMP2	3.8/0.002	-	-	−0.4/0.72	0.44	142.1
QTL+BMP2	5.9/0.0001	2.8/0.02	-	−1.0/0.35	0.61	136.3
HAO1+BMP2	4/0.003	-	0.3/0.75	−0.4/0.68	0.41	144.0
QTL+HAO1+BMP2	6.3/2.7×10-5	3.7/0.002	−2.2/0.05	−0.7/0.5	0.69	132.8

All details as per Table 1.

**Table 3:** Additional fecundity and bone morphometric traits correlated with *HAO1* and *BMP2* expression in bone samples.

	HAO1 expression in bone		
Trait	t-statistic	p-value	Adj R^2^
egg number (brooding)	2.6	0.02	0.88
total egg production (brooding)	2.8	0.02	0.89
broodiness	−2.4	0.03	0.89
comb mass	3.2	0.005	0.7
diaphyseal medullary bone	−3	0.01	0.63

	BMP2 expression in bone		
Trait	t-statistic	p-value	Adj R^2^
density of diaphyseal endosteal cavity	2.4	0.03	0.27
diaphyseal endosteal cavity area	−2.2	0.05	0.23
endosteal circumference	−2.3	0.04	0.24

Genotype (QTL) effect and body weight were included in each GLM.

## Discussion

In wild chicken populations, sexual ornaments are closely related with fitness traits [15]. We demonstrate that a block of two genes is controlling multiple aspects of the comb and bone allocation in the chicken, though the relative effects of both must be verified through functional assays. *BMP* genes have been shown to have numerous effects on bone physiology and increasing bone deposition [24], stimulating osteoblast proliferation and differentiation and stimulating bone formation. Cartilage is the precursor to all bone formation (the skeleton is first laid down cartilaginously, with this then becoming ossified [25]). This explains the link between the cartilage production required for comb growth and bone production physiology. *HAO1* on the other hand is a novel candidate for altering bone and cartilage deposition, to date principally shown to be expressed in the liver. Previous work has also highlighted its role in the liver metabolic pathways as a peroxisomal enzyme [26], and it is also implicated in lymphoblastic leukemia [27]. Peroxisomal enzymes feature heavily in the catabolism of long chain fatty acids [28] and are important for energy metabolism. Peroxisomal oxylate enzymes have also been linked to effects on calcium binding, through links with hereditary calcium oxalate kidney stone diseases (Primary Hyperoxaluria) [29], demonstrating potential interactions with calcium binding which may also affect the cartilage/bone allocation system.

Despite the pleiotropic effects seen between comb mass and bone allocation/egg production the overlap between comb mass QTL and selective sweeps may be due to two potential effects. On the one hand, if all comb mass loci exhibit pleiotropic effects on bone allocation or egg production, the strong selection for egg production will naturally cause this overlap to occur. However, an alternative explanation for this overlap is direct selection for comb mass itself is potentially occurring in these Layer breeds. This may be due to breeders intentionally selecting for large combs, realising they are a good indicator of egg production or indeed are a beneficial trait in of themselves. The comb is involved in heat regulation in the chicken [30], and therefore may also assist in survival in crowded domestic conditions.

The results also show that with one or both genes of this two-gene block, allelic variants can be seen to have effects on bone allocation, fecundity, brooding and comb growth. This highlights the importance of both pleiotropy and linkage in such systems. In this instance the extremely close linkage between the two genes in this block results in essentially a pleiotropic effect occurring from the alleles at these loci. This has important ramifications for understanding multiple complex trait interactions in domestication. Pleiotropy and close-linkage can work together to produce a pleiotropic effect on multiple divergent traits. Even here, the close linkage can be disrupted by recombination, enhancing the flexibility of the system. These results show that the pleiotropic core of domestication ‘modules’ (regions of the genome controlling multiple aspects of the domestication phenotype [7], [31]–[33]) contain both alleles with pleiotropic effect and extremely close linkage between QTL. Such a pattern of linkage and domestication modules are also seen in domesticated plants [31], [32], [34], [35], indicating such a system of loose and tight linkage is responsible for the domestication phenotype in a diverse range of taxa.

This study also potentially sheds light on the genetics of sexual selection. Although in the case of the domestic population it is no longer subject to sexual selection, the current artificial selection will be occurring on the pre-existing genetic architecture of the ancestral Red Junglefowl. In this instance, the natural selection constraints acting in the wild Junglefowl (which will limit the expensive investment of bone into fecundity in females to conserve longevity and fitness) can be ‘decoupled’ from the sexual ornament. The domestic population can potentially therefore show a counterpoint to the wild population in this regard. Interestingly, the observed pleiotropy ties in well with much of the previous work on sexual selection theory, as it has long been considered important in an accurate ornamental indicator signal [36] and in sexual selection theory in general [37]. Pleiotropy may also underlie the genetic covariation between traits that may in turn constrain the individual evolution of each trait independently [38], [39]. Similarly, condition dependence of a sexual ornament may also be due to pleiotropic effects [40]. Examples of the importance of pleiotropy in sexual ornaments can also be found by the overlap of multiple, functionally related, sexually selected traits. In *Drosophila* cuticular hydrocarbons [41] and wing song components [42], [43] have all been found to exhibit this overlapping architecture.

Pleiotropy can also have large consequences for long term phenotypic evolution and on complex traits in general [44]. Using global expression data, large-scale expression variation in between-sex [45] and breed [46]–[48] comparisons is at odds with the relatively modest QTL genetic architecture which is also detected in these comparisons (although power of detection is always a potential issue with QTL analysis). In these instances modules of genes (either pleiotropic or closely linked) once again appear to underlie the large-scale transcriptional changes. For example in a laboratory×wild cross of *S.cerevisiae* strains, genetic variation at a single gene was shown to have a major impact on global transcription [49], whilst recombinant inbred line studies using mice [50] and *Drosophila* [51] have also found modular effects of gene expression.

In the case of *Drosophila*, wild-derived inbred lines also show a high degree of modularisation in transcription [52]. It is perhaps pertinent that the modular patterns observed in domesticated animals is also seen once again in the wider variety of organisms previously listed. In the case of the chicken model presented here, the effect of domestication has been to decouple a sexual ornament from the limitations of natural selection. In wild populations, natural selection will act to limit the extreme allocation of calcium into medullary bone (which will increase short term reproductive gains at the expense of longer term survival), thereby preventing these alleles from becoming fixed in the population. During domestication this barrier has been removed (in fact this increased short-term fecundity is being actively selected for). However, by revealing the pleiotropic effects acting between the sexual ornament and the fitness trait, this does reveal how the indicator mechanism is maintained in the wild population, albeit not to the extreme levels seen in domestic populations.

## Materials and Methods

### Study Populations

Two separate crosses were conducted for this analysis, the first consisting of an F_2_ intercross between Obese Strain White Leghorn (WL-OS) chickens and a Red Junglefowl (RJF) population derived from a Swedish zoo population and maintained in Götala, Sweden (RJF-Götala). The second intercross was an eighth generation intercross between a line of selected White Leghorn (WL-L13) maintained from the 1960s and a population of RJF originally obtained from Thailand (RJF-T). This F_8_ advanced intercross line (AIL) is based on an F_2_ intercross that has been measured for comb mass and a variety of bone morphologies, see [8], [33], [53]. The WL-OS and WL-L13 cross have been separated since approximately 1955, so although they come from the same base population of White Leghorn chickens they have now had over 50 years of separation. Hereafter the abbreviation F_2_-L13 will be used for the F_2_ RJF-T/WL-L13 cross, F_8_-L13 for the F_8_ RJF-T/WL-L13 AIL, and F_2_-OS for the WL-OS/RJF-Götala F2 cross. In the case of the F_2_-OS cross, these were raised in a total of 4 batches at Götala research station of the Swedish University of Agricultural Sciences, Skara, Sweden. Chickens were maintained on standardised conditions and fed ad libitum, under a 12∶12 hour light/dark regime. Pens measuring 3 m×3 m were used for housing, with perches also provided. Individuals were culled at 200 days of age, with their combs surgically removed post-mortem and weighed and both femoral bones extracted. The F_8_-L13 cross used in this study was generated in 5 batches and reared at the research station of Linköping University, Sweden. Pens for these animals were 2 m×2 m and comprised of three separate levels, perches and food were once again supplied ad libitum. Animals were culled at 212 days of age, with the comb surgically removed post-mortem and weighed, and both femoral bones extracted.

### WL-OS/RJF-G

A total of 640 F_2_ individuals were used for the comb mass analysis, consisting of 308 males and 332 females. Of these, 543 of the individuals were also measured for a variety of bone morphology traits (245 males and 298 females). The WL-OS strain was originally isolated as a model of Hashimoto's thyroiditis, as they suffer from hypothyroidism and therefore require thyroxin to be provided in a food supplement (500 µg/kg food) (Levaxin Tablets, Nycomed AB, Sweden, were administered to all but one batch until 180 days of age). Although this strain tends to be slightly smaller than usual WL strains, they are still substantially larger, and with far greater sized combs, as compared to RJF. In addition, the degree of infiltration that had occurred in the F_2_ animals thyroid was also measured, and correlated with both comb mass and body weight. No significant correlations were found, and additionally the majority of the QTL discovered in this cross (7 of 11) all go in the expected direction, i.e. alleles of greater effect come from the WL, not RJF, line (with one of the three transgressive QTL also corroborated as transgressive in the other crosses as well). If any comb loci were caused by thyroiditis this would be expected to be the reverse, so we have good cause to consider these QTL are due to generic WL-strain based differences. Animals were culled at 200 days of age. The animal experiments were approved by the ethical committee for animal experiments in Göteborg, Sweden, no. 55-2005 and 233-2006, and by the ethical committee for animal experiments in Uppsala no. 2008-12-19, C321/8.

### WL-L13/RJF-T F_8_

A total of 447 F_8_ individuals were assayed for comb measurements (216 males and 231 females). These individuals were generated from a total of 107 families using 122 F_7_ individuals (63 females and 59 males). Average family size 4.7+/−3.1 (mean, standard deviation). These were the continuation of an inter-cross initially based on 3 WL females and one RJF male, which were then expanded into 811 F_2_ progeny and then maintained with at a population size of approximately 100 birds per generation until the F_7_ generation. Of the F_8_ individuals, a total of 41 males were used as the basis of a qPCR experiment using comb base tissue to check for candidate genes at the major chromosome 3 QTL locus at 15.7 Mb (12 RJF homozygotes, 15 WL homozygotes, 14 heterozygotes). These individuals came from two of the five batches used to produce the F_8_. Additionally, 20 females were used as the basis for a qPCR experiment using diaphyseal medullary bone (10 of each homozygote class, though one had no metaphyseal CT bone measurements, reducing the correlation with bone density to 19 individuals). The study was approved by the local Ethical Committee of the Swedish National Board for Laboratory Animals.

### WL-L13/RJF-T F_2_

A total of 352 males and 122 females were used in this analysis, with these QTL already detailed in [8], .

### Bone Phenotypic Measurements

The right femoral bone of each F_2_OS and F_8_L13 individual was measured using a Peripheral Quantitative Computerised Tomography (pQCT) machine (Stratec XCT – Research SA machine, Stratec Medizintechnik, Germany), with two sections taken at 6% (metaphyseal) and two at 50% (diaphyseal) of the total femoral length. Cortical bone was measured using the CORTMODE1 setting, with a density threshold of >1000 mg/cm^3^. Cortical measures were cortical area, cortical bone content, cortical thickness and cortical density. Medullary measures were recorded using the PEELMODE2 function, using an inner threshold of 1000 mg/cm^3^ to separate cortical from medullary bone and gives total density, total bone content and total area of the endosteal cavity measures. An additional inner threshold of 150 mg/cm^3^ in a combination of two PEELMODE2 gives the medullary area, medullary density and medullary bone content.

### Fecundity Phenotypic Measures

Two separate fecundity trials were performed. Initially birds were housed individually and eggs were collected daily over a two-week period. The second trial was performed immediately after the first and was identical except birds were given two dummy eggs to incubate and were allowed to keep all eggs laid over a two-week period. At the end of each trial, number of eggs produced, total weight of eggs produced and mean egg weight were recorded. Chickens which are actively brooding (incubating) their eggs will stop producing eggs when a clutch size of around 6–8 eggs has been produced, whilst domestic layers will continually produce eggs and never go into such brooding behaviour. Therefore one method for ascertaining if an individual is brooding will be to deduct the total number of eggs produced in the first trial from the number of eggs produced in the second trial. Negative values therefore indicate that an individual is decreasing egg production when allowed to build up a clutch.

### Phenotypic Correlations

To analyse phenotypic correlations between comb mass and bone characteristics in the F_2_OS and F_8_L13 crosses, a GLM was fitted for each individual bone trait measured and included batch and sex fixed effects and weight at slaughter as a covariate. All significantly correlated bone measures were then combined into a global model. The significance values of each measure was then ascertained in this global model.

### Clustering Analysis

The clustering test was performed using a permutation test based on the total length of the chicken genome (1.09 Gb), which then had a number of regions equal to the number of QTL detected in the F_2_OS and F_8_L13 cross randomly distributed along it. The size of these regions was equal to the average C.I. of QTL from the F_2_-OS cross (15 Mb) and the F_8_L13 cross (5 Mb), and tested against the observed number of overlaps between the F_2_OS and F_8_L13 (6). The F_2_L13 cross was not used in this analysis, as 4 of the 5 QTL detected in this cross were strongly replicated in the F_8_L13 and their inclusion could artificially inflate the degree of replication observed between the two different cross populations. This was repeated 1000 times, with the number of overlaps recorded each time used to generate a significance value. A similar procedure was used to predict the probability of selective sweeps occurring within the overlap regions. In this instance the total number of sweeps detected (133) and the six overlap regions were used, based against the observation that 13 sweeps were observed within the six 6 Mb overlap intervals. When calculating the overlap regions, an extra 1 Mb was added to the region size upstream and downstream, in case using only the overlap between the OS and L13 crosses gave an artificially small region.

### Genotyping and QTL Analysis

DNA preparation for both crosses was performed by Agowa GmbH (Berlin, Germany), using standard salt extraction. A total number of 347 SNPs and 20 microsatellite markers were used for the OS cross, with the marker map generated using Crimap [54], covering ∼2535 cM, with an average marker spacing of ∼7.5 cM. For the F_8_L13 cross, 652 SNP markers were used to generate a map of length ∼8760 cM, with an average marker spacing of ∼15 cM. QTL analysis was performed using QTL Express (http://qtlcap.ed.ac.uk), qxpak v2.16 [55] and R/Qtl [56] for the standard interval mapping and epistatic analyses. Interval mapping was performed using additive and additive+dominance models. Batch and sex were always included in the model as a fixed effect, whilst bodyweight at slaughter was included as a covariate. To account for a particular QTL varying between the sexes, a sex-interaction analysis was also performed.

### Significance Thresholds

Significance thresholds for both crosses were calculated using permutation tests [57], [58], however these values differed greatly between the two study populations due to the massively increased number of recombinations in the F_8_L13 population (as reflected in the inflated map size) and also to account for possible family effects in this population. A suggestive significance level of a genome-wide 20% threshold was used (mainly due to being more conservative than the standard suggestive threshold suggested by Lander and Kruglyak [59]). For the F_2_OS cross, additive+dominance effects had a suggestive threshold of around ∼3.0 and a significant threshold of ∼3.9, depending on the trait, whilst additive+dominance sex interaction models had a suggestive threshold of ∼3.8 LOD and a significant threshold of ∼4.6 LOD. In the case of the comb analysis, a single point threshold was used where a QTL had been indicated in the F_8_L13 or F_2_L13 mapping populations at a locus, but where a significant or suggestive QTL in the F_2_OS population was not immediately apparent. 5% single point threshold for additive only model was LOD ∼1.4, whilst for an additive+dominance model this was LOD ∼2.0. Confidence intervals (C.I.) for QTL were calculated using a 1.8 LOD drop method (i.e. where the LOD score on either side of the peak decreases by 1.8 LOD). The nearest marker to this 1.8 LOD decrease is then used to give the C.I. in megabases (the use of the physical location of markers allows a direct comparison between the different crosses). The use of a 1.8 LOD drop has been shown to most reliably give an accurate 95% confidence interval for an intercross type population [60].

Thresholds for an AIL are potentially problematic, as the family structure can cause inflated LOD scores, resulting in false positive results. To avoid this initially, we used a large number of families (107) to generate the total number of individuals, to break down this sub-structure as much as possible. For instance, if only one offspring were used per family, there would be no structure and the population would function exactly as recombinant inbred lines [61]. To check if family was a significant factor on the comb trait, a GLM consisting of sex, batch, family and weight was used to predict comb weight. In this instance, only one family out of 107 was significant (p = 10^−6^), and consisted of two large males. Furthermore to generate the significance thresholds we also used a family factor included in the permutation model, which resulted in greater thresholds. The threshold without family as a factor was LOD 3.4 for a suggestive threshold and LOD 4.15 for a significant threshold. With family included as a cofactor, a suggestive threshold was 4.55 and a significant threshold was 5.4 was obtained.

### Gene Expression Measurements

Comb base RNA was isolated with Ambion TRI reagent (Applied Biosystems, Carlsbad, CA, USA), following the manufacturer's protocol. After removal of the comb, a part of the forehead was frozen in liquid nitrogen. Frozen comb base tissue was removed with a razor and disrupted with a hammer and bag. Samples were homogenized on a FastPrep-24 instrument (MP Biomedicals, Solon, OH, USA) in tubes with TRI and ceramic beads (Lysing matrix D, MP Biomedicals).

First strand cDNA for qPCR was made with Fermentas (St. Leon-Rot, Germany) RevertAid Reverse Transcriptase, using 10 mM dNTPs, RiboLock nuclease inhibitor, and oligo(dT)_18_ primer (Thermo Fisher Scientific, Freemont, CA, USA), according to the manufacturer's protocol. qPCR was performed with Maxima SYBR Green qPCR mastermix (Thermo Fischer Scientific) in 15 µl reactions with 0.3 M of each oligonucleotide primer on a Rotor-Gene 6000 real-time cycler (Corbett Research, Cambridge, UK). The PCR program consised of a 10 min activation step at 95°C, followed by 40 cycles of 15 s at 95°C, and 1 minute at 60°C. After cycling, products were melted by ramping the temperature from 72°C to 95°C. qPCR data was analysed with the comparative ΔΔCt method [62]. Target gene threshold cycle values were normalized by subtracting the geometric mean value of three reference genes, *β2 microglobulin*, *TATA box binding protein*, and *RNA polymerase II subunit C1*. A batch effect (due to individuals coming from two of the five batches comprising the cross) was included in the General Linear Model analysis, as well as weight at slaughter for all individuals, when comb mass was included in the model.

### Linear Models and Expression

To ascertain the effects of differential gene expression from the two candidate genes in the QTL interval on the phenotypic trait, the gene expression levels (described above) were used in a linear model. The initial model to test gene expression was y (comb mass) = mean + batch + weight at slaughter + expression level (called either *HAO1* or *BMP2* in the model column in Tables 1 and 2). To test the effect of this on genotype an additional QTL genotype factor was added (model *HAO1* + QTL and *BMP2* + QTL in Tables 1 and 2). One measure of causality is that the additional of a candidate gene expression covariate should decrease the QTL genotype effect in this model, therefore these models were then checked against the model y = mean + batch + weight at slaughter + QTL genotype (QTL only model in Tables 1 and 2), to observe the drop in QTL significance. Additionally, two further models were also checked. These were y = mean + batch + weight at slaughter + *BMP2* expression +* HAO1* expression, and y = mean + batch + weight at slaughter + *BMP2* expression + *HAO1* expression+QTL genotype. These were fitted to check for the potential of gene interactions between both candidates affecting the trait.

Linear models were also fitted to test the association between *HAO1* and *BMP2* levels with the other comb mass QTL identified. Initially, a basic model of y (gene expression level) = mean + batch + weight at slaughter + QTL genotype was fitted for each QTL separately. All significant QTL were then combined into a single model (y = mean + batch + QTL1 + QTL2, etc) to test for the global combined significance. This global model was then compared to the base model with only the chromosome 3 QTL genotype included.

Linear models were also used to test the effects of *HAO1* and *BMP2* expression levels on other related fecundity and bone morphometric traits. In this case, a model of y (trait of interest) = mean + weight at slaughter + QTL genotype (chromosome 3) + *HAO1/BMP2* expression level was fitted.

## Supporting Information

Figure S1 Genetic map of the F_8_-L13 cross (autosomes only), with the location of F_8_-L13, F_2_-L13 and F_2_-OS QTL all marked on. Regions with more than one QTL from a cross (i.e. clusters of replicated QTL between crosses) are marked with red circles on the map.
(DOCX)

Table S1 QTL locations (in Mb) and effect sizes for the three separate crosses. ‘Model’ refers to whether an additive, additive+dominance, or additive+dominance sex interaction model was used to detect that QTL. R^2^ gives the proportion of variance explained by that QTL. The significance of each QTL is given as a LOD score, and the position of each QTL is given in megabases (Mb). The additive and dominance effects of each QTL are given in the ‘Male/combined’ additive and dominance columns in the case of QTL detected using standard additive and additive+dominance models, with the ‘Female additive and dominance’ columns then blank. In the case of QTL detected using an additive+dominance sex interaction model, the male additive+dominance effects are given first in the ‘male additive+dominance’ columns, and the female additive+dominance effects are given in the second ‘female additive+dominance’ columns. The confidence interval (C.I.) of each QTL is calculated using a 1.8 LOD drop and then the closest marker outside this interval is then selected to determine genomic position. This range is then shown in the ‘CI’ column.
(DOCX)

Table S2 Positions of QTL clusters in Mb and their overlap with selective sweeps. The locations of every sweep are given in Mb, followed by ‘LR’ to signify a layer-specific selective sweep, and ‘AD’ to signify an all-domestic selective sweep.
(DOCX)

Table S3 Combined GLM coefficients and significance for comb mass and bone allocation in the F_8_L13 and F_2_OS crosses. Results show the combined models for bone traits modelled on comb mass. In addition to the bone traits, batch and bodyweight were also included in the model (i.e. the models used were: comb mass∼batch+bodyweight+bone covariates). Metaphysis is abbreviated with met., diaphysis with diaph. for the bone variable names.
(DOCX)
