# Supplementary material for: A Sexual Ornament in Chickens Is Affected by Pleiotropic Alleles at HAO1 and BMP2, Selected during Domestication
Source: PLoS Genet. 2012 Aug 30;8(8):e1002914. doi: 10.1371/journal.pgen.1002914 (PMC3431302; doi:10.1371/journal.pgen.1002914)
Supplement: Figure S1 — Genetic map of the F8-L13 cross (autosomes only), with the location of F8-L13, F2-L13 and F2-OS QTL all marked on. Regions with more than one QTL from a cross (i.e. clusters of replicated QTL between crosses) are marked with red circles on the map. (DOCX) [file pgen.1002914.s001.docx]

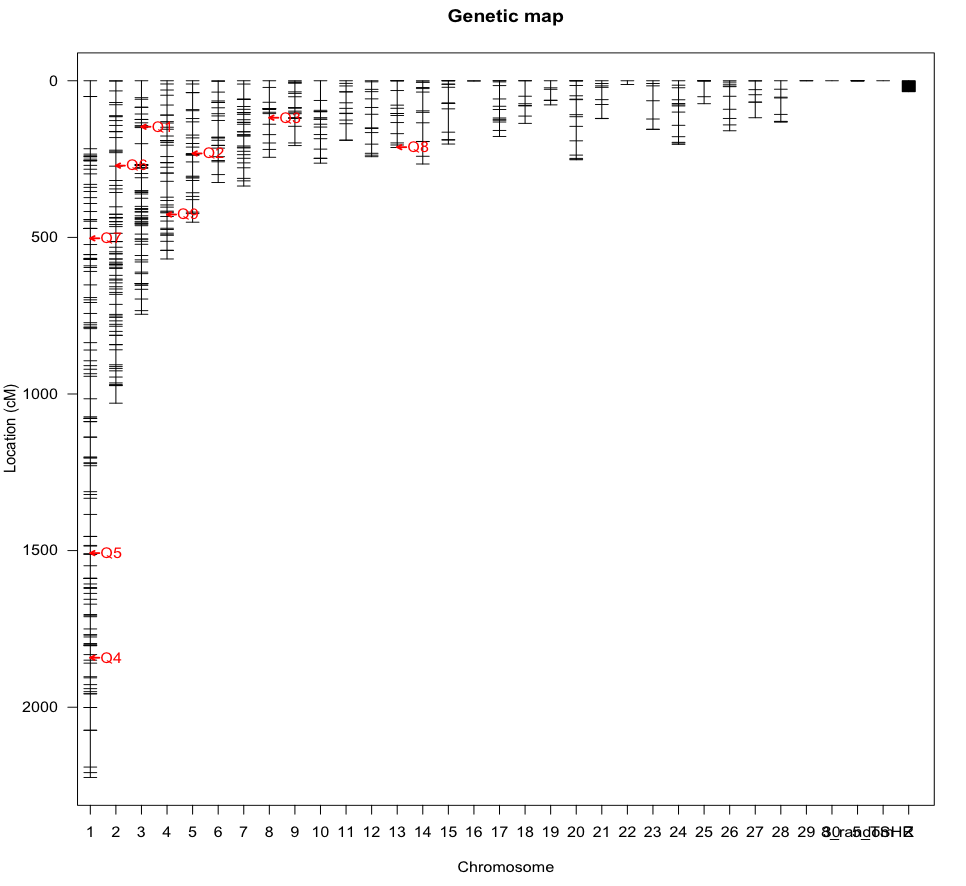
Supplementary figure 1

**Q4**

**Q10**

**Q9**

**Q8**

**Q7**

**Q6**

**Q5**

**Q5**

**Q4**

**Q3**

**Q2**

**Q1**

**Q1**

**Q3**

**Q2**

Comb QTL:

F_2_-L13 = 5 QTL

F_2_-OS = 10 QTL

F_8_-L13 = 9 QTL

Overlap of detected QTL between the three different crossing populations P=0.0016

F_8_-L13 = Red

F_2_-L13 = Blue

F_2_-OS = Black
