# Supplementary material for: A Sexual Ornament in Chickens Is Affected by Pleiotropic Alleles at HAO1 and BMP2, Selected during Domestication
Source: PLoS Genet. 2012 Aug 30;8(8):e1002914. doi: 10.1371/journal.pgen.1002914 (PMC3431302; doi:10.1371/journal.pgen.1002914)
Supplement: Table S1 — QTL locations (in Mb) and effect sizes for the three separate crosses. ‘Model’ refers to whether an additive, additive+dominance, or additive+dominance sex interaction model was used to detect that QTL. R2 gives the proportion of variance explained by that QTL. The significance of each QTL is given as a LOD score, and the position of each QTL is given in megabases (Mb). The additive and dominance effects of each QTL are given in the ‘Male/combined’ additive and dominance columns in the case of QTL detected using standard additive and additive+dominance models, with the ‘Female additive and dominance’ columns then blank. In the case of QTL detected using an additive+dominance sex interaction model, the male additive+dominance effects are given first in the ‘male additive+dominance’ columns, and the female additive+dominance effects are given in the second ‘female additive+dominance’ columns. The confidence interval (C.I.) of each QTL is calculated using a 1.8 LOD drop and then the closest marker outside this interval is then selected to determine genomic position. This range is then shown in the ‘CI’ column. (DOCX) [file pgen.1002914.s002.docx]

| Cross | Trait | chr | position(Mb) | model | LOD | r-2 | Male/ combined  add±s.e | Male/ combined  dom±s.e. | Female  add±s.e | Female  dom±s.e. | CI(Mb) |
| --- | --- | --- | --- | --- | --- | --- | --- | --- | --- | --- | --- |
| F_2_-OS | comb mass | 1 | 25.5 | a+d s.i. | 3.5 | 0.03 | 0.55±0.41 | 2.18±0.59 | 0.19±0.40 | 0.26±0.59 | 15.8-32.9 |
| F_8_-L13 | comb mass | 1 | 35.5 | a+d s.i. | 6.2 | 0.03 | 2.16±2.02 | -15.0±3.9 | - | - | 29-36.7 |
| F_2_-L13 | female comb mass | 1 | 38 | a+d | 5.6 | 0.14 | 0.55±0.13 | -0.20±0.19 | - | - | 30-50.5 |
| F_2_-L13 | female comb mass | 1 | 71.2 | a | 2.4 | 0.09 | 0.34±0.11 | -0.17±0.16 | - | - | 62.2-79.9 |
| F_8_-L13 | comb mass | 1 | 123 | a+d | 11.0 | 0.08 | 1.81±0.52 | -3.60±0.80 | - | - | 120-127 |
| F_8_-L13 | comb mass | 1 | 172 | a+d s.i. | 7.4 | 0.03 | -5.99±1.52 | 6.04±2.13 | - | - | 166.9-177.2 |
| F_2_-L13 | combined comb mass | 1 | 173.4 | a | 2.7 | 0.03 | -1.83±0.51 | - | - | - | 147.6-182 |
| F_2_-OS | comb mass | 1 | 183.7 | a+d s.i. | 6.1 | 0.05 | -1.38±0.42 | -2.76±0.65 | -0.01±0.40 | -0.92±0.62 | 152-189.7+ |
| F_8_-L13 | comb mass | 2 | 33.4 | a+d | 11.2 | 0.08 | -1.85±0.43 | -1.62±0.56 | - | - | 28.9-38.5 |
| F_2_-L13 | female comb mass | 2 | 93.2 | a | 2.4 | 0.09 | -0.37±0.11 | - | - | - | 68-115 |
| F_2_-OS | comb mass | 2 | 135.9 | a+d s.i. | 4.0 | 0.03 | -0.31±0.40 | -2.50±0.59 | -0.20±0.39 | 0.21±0.58 | 87.6-141.6 |
| F_2_-L13 | male comb mass | 3 | 14 | a+d | 9.5 | 0.11 | -4.35±0.68 | -2.36±1.13 | - | - | 6.6-15.6 |
| F_8_-L13 | comb mass | 3 | 16 | a+d s.i. | 37.7 | 0.22 | -10.9±1.0 | -3.22±1.50 | - | - | 15.6-16.0 |
| F_2_-OS | comb mass | 3 | 13-16 | a | 1.5 | 0.02 | -0.49±0.39 | -1.57±0.58 | 0.05±0.39 | -0.03±0.56 | - |
| F_2_-OS | comb mass | 4 | 51.3 | a+d s.i. | 8.0 | 0.06 | 2.44±0.41 | 0.32±0.61 | 0.49±0.39 | -0.18±0.59 | 39.2-60.3 |
| F_8_-L13 | comb mass | 4 | 68 | a+d s.i. | 5.4 | 0.03 | -2.25±1.71 | -5.04±2.22 | - | - | 64.8-71.8 |
| F_8_-L13 | comb mass | 5 | 32.70 | a+d | 4.7 | 0.03 | 0.70±0.35 | -1.87±0.47 | - | - | 29.6-36 |
| F_2_-OS | comb mass | 7 | 34.6 | a+d s.i. | 4.8 | 0.04 | -1.68±0.39 | -1.06±0.59 | -0.25±0.38 | 0.47±0.57 | 27.8-36.5 |
| F_2_-OS | comb mass | 8 | 14.4 | a+d s.i. | 2.5 | 0.02 | -1.23±0.38 | 0.49±0.54 | 0.09±0.37 | 0.28±0.53 | 4.5-22.1 |
| F_8_-L13 | comb mass | 8 | 20.0 | a+d s.i. | 9.5 | 0.05 | -4.24±1.59 | 7.82±2.70 | - | - | 19.60-21.60 |
| F_2_-L13 | male comb mass | 8 | 23.9 | a | 2.8 | 0.04 | -2.54±0.70 | -0.47±1.20 | - | - | 4.1-29.9+ |
| F_2_-OS | comb mass | 10 | 17.9 | a+d s.i. | 4.8 | 0.04 | -1.77±0.43 | 1.24±0.71 | -0.55±0.44 | -0.47±0.69 | 12.8-22 |
| F_2_-OS | comb mass | 13 | 9 | a+d s.i. | 5.9 | 0.04 | -1.95±0.41 | -1.47±0.60 | -0.07±0.40 | 0.05±0.59 | 2.4-11.2+ |
| F_8_-L13 | comb mass | 13 | 17 | a+d s.i. | 5.9 | 0.03 | -4.52±1.13 | -4.47±1.54 | - | - | 15.1-17.9+ |
| F_2_-OS | comb mass | 17 | 3.9 | a+d s.i. | 5.2 | 0.04 | 0.95±0.41 | 2.55±0.61 | -0.16±0.41 | 0.25±0.59 | 0-7.2 |
| F_2_-OS | comb mass | 28 | 0.4 | a+d s.i. | 5.0 | 0.04 | -2.05±0.46 | -1.23±0.70 | -0.16±0.42 | -0.20±0.67 | 0-3.6+ |

Table S1. QTL locations (in Mb) and effect sizes for the three separate crosses. ‘Model’ refers to whether an additive, additive+dominance, or additive+dominance sex interaction model was used to detect that QTL. R^2^ gives the proportion of variance explained by that QTL. The significance of each QTL is given as a LOD score, and the position of each QTL is given in megabases (Mb). The additive and dominance effects of each QTL are given in the ‘Male/ combined’ additive and dominance columns in the case of QTL detected using standard additive and additive+dominance models, with the ‘Female additive and dominance’ columns then blank. In the case of QTL detected using an additive+dominance sex interaction model, the male additive+dominance effects are given first in the ‘male additive+ dominance’ columns, and the female additive+dominance effects are given in the second ‘female additive+dominance’ columns. The confidence interval (C.I.) of each QTL is calculated using a 1.8 LOD drop and then the closest marker outside this interval is then selected to determine genomic position. This range is then shown in the ‘CI’ column.
