# Supplementary material for: A Sexual Ornament in Chickens Is Affected by Pleiotropic Alleles at HAO1 and BMP2, Selected during Domestication
Source: PLoS Genet. 2012 Aug 30;8(8):e1002914. doi: 10.1371/journal.pgen.1002914 (PMC3431302; doi:10.1371/journal.pgen.1002914)
Supplement: Table S2 — Positions of QTL clusters in Mb and their overlap with selective sweeps. The locations of every sweep are given in Mb, followed by ‘LR’ to signify a layer-specific selective sweep, and ‘AD’ to signify an all-domestic selective sweep. (DOCX) [file pgen.1002914.s003.docx]

| chr | bp | #sweeps | locations in Mb | region size (Mb) | #genes |
| --- | --- | --- | --- | --- | --- |
| 1 | 30-34Mb | 5 | 29.72–29.80 LR, 29.82–29.90 LR, 34.26–34.30 LR, 34.32–34.40 LR, 34.62–34.66LR | 6 | 6 |
| 1 | 167-178Mb | 2 | 168.54–168.58 LR, 179.66–179.70 LR | 13 | 2 |
| 3 | 15.6Mb | 1 | 15.60–15.68 LR | 2 | 1 |
| 4 | 60-65Mb | 4 | 58.46–58.56 AD, 64.56–64.60 AD,:64.76–64.80 AD, 65.06–65.10 AD | 7 | 13 |
| 8 | 20-22Mb | 1 | 19.72–19.78 AD | 4 | 3 |
| 13 | 15-18Mb | 0 |  | 5 |  |

Table S2. Positions of QTL clusters in Mb and their overlap with selective sweeps. The locations of every sweep are given in Mb, followed by ‘LR’ to signify a layer-specific selective sweep, and ‘AD’ to signify an all-domestic selective sweep.
