# Supplementary material for: A Sexual Ornament in Chickens Is Affected by Pleiotropic Alleles at HAO1 and BMP2, Selected during Domestication
Source: PLoS Genet. 2012 Aug 30;8(8):e1002914. doi: 10.1371/journal.pgen.1002914 (PMC3431302; doi:10.1371/journal.pgen.1002914)
Supplement: Table S3 — Combined GLM coefficients and significance for comb mass and bone allocation in the F8L13 and F2OS crosses. Results show the combined models for bone traits modelled on comb mass. In addition to the bone traits, batch and bodyweight were also included in the model (i.e. the models used were: comb mass∼batch+bodyweight+bone covariates). Metaphysis is abbreviated with met., diaphysis with diaph. for the bone variable names. (DOCX) [file pgen.1002914.s004.docx]

| Cross | trait | Estimate + s.e. | P-value |
| --- | --- | --- | --- |
| F8 female  N= 219 | met. medullary density (mg/cm3) | -8x10^-3^ +/- 3x10^-3^ | 0.002 |
| F8 female | diaph. medullary density (mg/cm3) | 5x10^-3^ +/- 2x10^-3^ | 0.004 |
| F8 female | diaph. cortical thickness (mm) | 6 +/- 1 | 3x10^-9^ |
| F8 female | diaph. cortical density (mg/cm3) | 1x10^-2^ +/- 5x10^-3^ | 0.01 |
| F8 male  N= 214 | met. total bone content (mg/mm) | 2 +/- 6x10^-1^ | 0.002 |
| F8 male | met. total density (mg/cm3) | 5x10^-2^+/- 2x10^-2^ | 0.01 |
| F8 male | met. medullary bone content (mg/mm) | 2 +/- 5x10^-1^ | 0.0004 |
| F8 male | diaph. cortical density (mg/cm3) | 0.2+/- 4x10^-2^ | 2x10^-5^ |
| OS female n= 283 | diaph. medullary area (mm2) | 0.2 +/- 0.03 | 4x10^-7^ |
| OS female | diaph. cortical area (mm2) | -2 +/- 0.4 | 2x10^-4^ |
| OS female | diaph. cortical bone content (mg/mm) | 0.9 +/- 0.3 | 0.007 |
| OS female | diaph. cortical thickness (mm) | 7 +/-2 | 0.007 |
| OS female | met. total bone density (mg/cm3) | -5x10^-3^ +/- 1x10^-3^ | 9x10^-5^ |
| OS female | met. medullary area (mm2) | 1x10-2 +/- 6x10-3 | 0.07 |
| OS male  n= 242 | met. medullary density (mg/cm3) | -0.2 +/- 0.09 | 0.07 |
| OS male | met. medullary area (mm2) | 0.2 +/- 0.05 | 0.003 |
| OS male | met. total bone density (mg/cm3) | 0.2 +/- 0.08 | 0.06 |
| OS male | diaph. cortical bone content (mg/mm) | 0.6 +/- 0.2 | 0.005 |

Table S3. Combined GLM coefficients and significance for comb mass and bone allocation in the F_8_L13 and F_2_OS crosses. Results show the combined models for bone traits modelled on comb mass. In addition to the bone traits, batch and bodyweight were also included in the model (i.e. the models used were: comb mass~batch+bodyweight+bone covariates). Metaphysis is abbreviated with met., diaphysis with diaph. for the bone variable names.
